# Functional and Structural Properties of Dentate Granule Cells with Hilar Basal Dendrites in Mouse Entorhino-Hippocampal Slice Cultures

**DOI:** 10.1371/journal.pone.0048500

**Published:** 2012-11-07

**Authors:** Denise Becker, Laurent Maximilian Willems, Matej Vnencak, Nadine Zahn, Gerlind Schuldt, Peter Jedlicka, Nicola Maggio, Thomas Deller, Andreas Vlachos

**Affiliations:** 1 Institute of Clinical Neuroanatomy, Neuroscience Center, Goethe-University Frankfurt, Frankfurt, Germany; 2 Talpiot Medical Leadership Program, Department of Neurology and J. Sagol Neuroscience Center, The Chaim Sheba Medical Center, Tel HaShomer, Israel; University of Nebraska Medical Center, United States of America

## Abstract

During postnatal development hippocampal dentate granule cells (GCs) often extend dendrites from the basal pole of their cell bodies into the hilar region. These so-called hilar basal dendrites (hBD) usually regress with maturation. However, hBDs may persist in a subset of mature GCs under certain conditions (both physiological and pathological). The functional role of these hBD-GCs remains not well understood. Here, we have studied hBD-GCs in mature (≥18 days in vitro) mouse entorhino-hippocampal slice cultures under control conditions and have compared their basic functional properties (basic intrinsic and synaptic properties) and structural properties (dendritic arborisation and spine densities) to those of neighboring GCs without hBDs in the same set of cultures. Except for the presence of hBDs, we did not detect major differences between the two GC populations. Furthermore, paired recordings of neighboring GCs with and without hBDs did not reveal evidence for a heavy aberrant GC-to-GC connectivity. Taken together, our data suggest that in control cultures the presence of hBDs on GCs is neither sufficient to predict alterations in the basic functional and structural properties of these GCs nor indicative of a heavy GC-to-GC connectivity between neighboring GCs.

## Introduction

Among principal neurons within the hippocampus mature dentate granule cells (GCs) can be identified by their characteristic morphology. Their cell bodies, which are densely packed in the granule cell layer, extend dendrites into the molecular layer while an axon usually emerges at the basal pole of the soma contacting hilar mossy cells and CA3 pyramidal neurons (for review see [1], [2]). Immature GCs show less complex and much shorter dendritic trees compared to adult GCs [3] and at a certain developmental stage dendrites may be observed which emerge from basal portions of the soma and reach into the hilar region (for review see [4], [5]). While the majority of these hilar basal dendrites (hBD) regresses with maturation [5], [6], a subset of mature GCs shows persisting hBDs under physiological conditions, both in vitro [7] as well as in vivo [8]. Of note, in primates it has been estimated that ∼10% of all GCs exhibit basal dendrites [9], [10]. Although these neurons do not appear to be rare, data on functional and structural properties of mature hBD-GCs under control conditions remain scarce [11].

Notably, hBD-GCs appear to be more numerous under pathological conditions, such as hypoxia-ischemia [12], experimentally induced epilepsy [13], and pharmacoresistant temporal lobe epilepsy of humans [14]. This is in line with experimental evidence, which suggests that neuronal hyperactivity can stabilize basal dendrites on immature GCs [7]. These findings have led to the hypothesis, that hBD-GCs could be involved in the pathogenesis of epilepsy in the dentate gyrus. On the one hand it has been proposed that hBDs could cause hyperexcitability in the hippocampus, e.g., by promoting aberrant GC-to-GC connectivity in the dentate gyrus [5], [15]. On the other hand, it has been suggested that mossy fibers form synapses on basal dendrites [13] in order to compensate for the loss of hilar neurons due to epilepsy [16]. Thus, further characterization of mature hBD-GCs is needed to better understand their role in physiology and pathology.

Organotypic entorhino-hippocampal slice cultures are suitable tools to study the development and maturation of GCs with basal dendrites [7], [17]. In mature slice cultures hBD-GCs are frequently found, even under control conditions, and their functional and structural properties can be directly compared to those of neighboring GCs lacking hBDs. Here, we have used ≥18 days in vitro old mouse entorhino-hippocampal slice cultures to address the question whether hBD-GCs differ in their properties from GCs without hBDs. Our data suggest that the two populations of GCs do not show major differences in their basic functional and structural properties (except for the presence of hBDs) in this in vitro setting. Furthermore, we did not observe evidence for a strong aberrant GC-GC connectivity, suggesting that the presence of hBDs on GCs *per se* is not sufficient to predict a pathological, i.e., hyperexcitable dentate gyrus.

## Results

**Figure 1:**
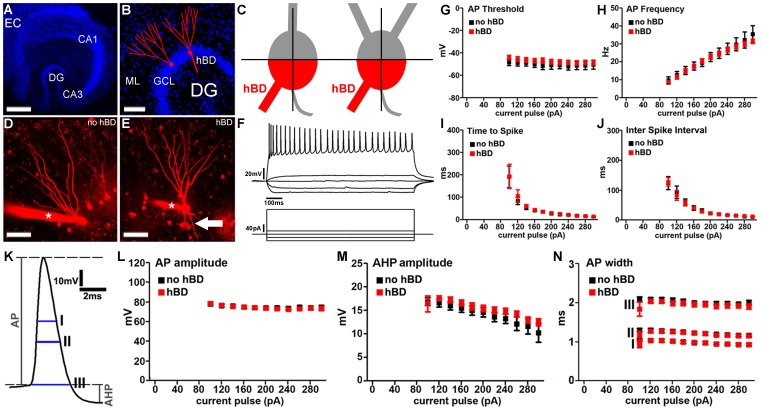
Basic intrinsic cellular properties of dentate granule cells with hilar basal dendrites. (A) Overview of a mature entorhino-hippocampal slice culture (blue, TOPRO nuclear stain; EC, entorhinal cortex; DG, dentate gyrus; CA1, hippocampal subfield CA1). Scale bar: 500 µm. (B) Dentate gyrus of a mature entorhino-hippocampal slice culture shown at higher magnification. Schematic representations of dentate granule cells. Granule cell somata are located in the granule cell layer (GCL) while granule cell dendrites extend into the molecular layer (ML). A subset of mature dentate granule cells may exhibit additional dendrites, which emerge from the basal portion of the soma and extend into the hilar region (hilar basal dendrites; hBDs; blue, TOPRO nuclear stain). Scale bar: 100 µm. (C) Hilar basal dendrites (hBDs) were defined using the following objective criteria. The granule cell soma was divided into quarters by connecting the origin of the axon with the apical pole of the soma and by an orthogonal centerline trough this line. The two quarters next to the origin of the axon were called basal quarters (red). Dendrites emerging from the basal quarters, reaching into the hilar region and not crossing the centerline were considered as hBDs. (D, E) 2D-projected confocal image stacks of dentate granule cells filled with Alexa568 (red). Asterisk indicates patch electrode. Arrow points to a hBD. Scale bar: 50 µm. (F) Sample traces showing input-output curves of a hBD-GC. Voltage traces (top) in response to a 1 sec current pulse. Protocol is shown underneath the traces. (G–J) Granule cells with and without hBDs were indistinguishable in their (G) actionpotential (AP)-threshold, (H) AP frequency, (I) time to first spike or (J) inter spike interval (n = 9 GCs without hBDs and n = 12 GCs with hBDs; in 7 cultures). (K–N) Evaluation of action potential properties did not show a significant difference between the two GC populations in (L) AP-amplitude, (M) afterhyperpolarization (AHP) amplitude or AP width (N) measured at three different positions (indicated with I, II and III in panel K).

Mature (≥18 days in vitro, div) entorhino-hippocampal slice cultures (Figure 1A) were used to compare functional and structural properties of cultured GCs with and without hBDs (Figure 1B). Dendrites extending from the basal pole of the GC soma into the hilar region and not crossing an orthogonal centerline through the connecting line between the basal and apical pole of the GC soma (i.e., not entering the molecular layer; cf. [17]) were considered to be hBDs (Figure 1C). In our experiments neurons were patched and filled with Alexa568 or Alexa488. This allowed us to readily identify GCs with hBDs based on the above mentioned criteria and thus to distinguish between the two cell populations of interest in confocal image stacks prior to recordings (Figure 1D, E).

### Basic Input-output Properties of GCs with hBDs

To examine whether GCs with hBDs exhibit major changes in their intrinsic cellular properties, input-output properties were determined from the two groups of neurons in a whole-cell configuration. As shown in Figure 1F current pulses were applied to GCs for 1 sec. ranging from −80 pA to +300 pA (ΔI = 20 pA). The mean resting membrane potential was not significantly different between the two groups of GCs (no hBDs: −77.4±3.2 mV; hBDs: −79.8±1.8 mV). GCs with and without hBDs were indistinguishable in their action potential (AP)-threshold (Figure 1G), in AP-frequency (Figure 1H), in their mean time to first spike (Figure 1I), as well as in their inter spike interval (ISI, Figure 1J) and ISI-adaptation (data not shown). Evaluations of AP properties (Figure 1K) revealed no significant change of AP-amplitude (Figure 1L), afterhyperpolarization (AHP) amplitude (Figure 1M) and width of the first AP (Figure 1N) in hBD-GCs. The two groups were indistinguishable in AP-adaptation and AHP-adaptation as well (data not shown). Taken together, these results showed that hBD-GCs of mature entorhino-hippocampal slice cultures do not show major differences in their basic intrinsic cellular properties compared to GCs without hBDs.

### Excitatory Synaptic Strength of hBD-GCs

**Figure 2:**
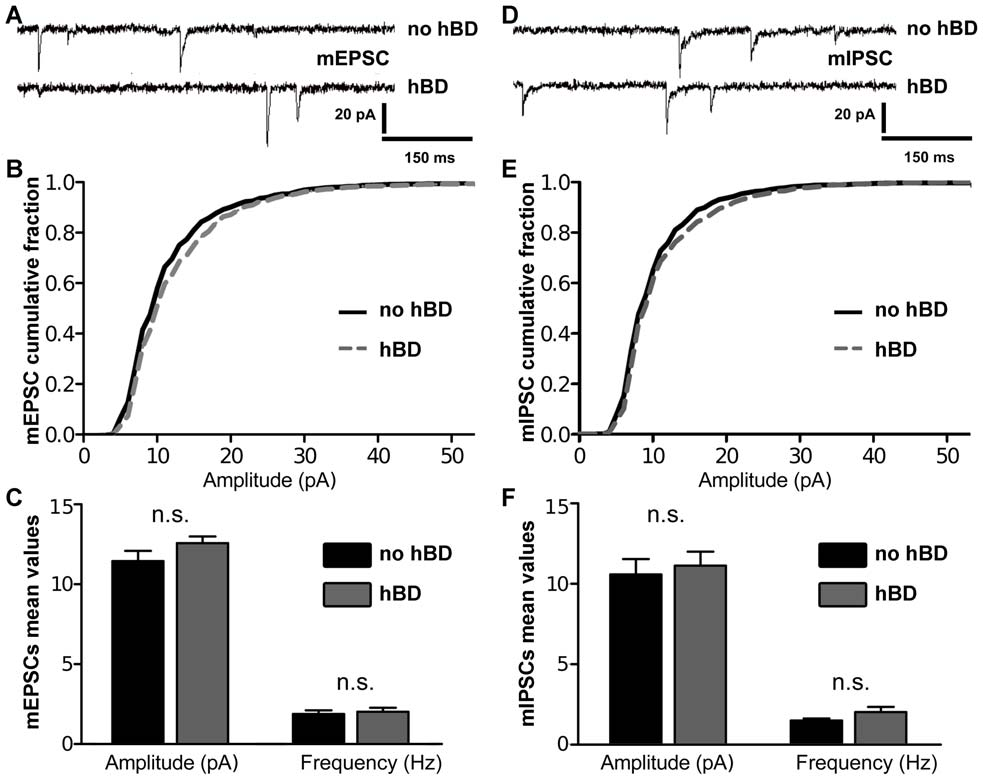
Excitatory and inhibitory synaptic strength of dentate granule cells with hilar basal dendrites. (A–C) Sample traces of miniature excitatory postsynaptic currents (mEPSC). Cumulative distribution of the mEPSC amplitudes and mean values of mEPSC amplitude and frequency (n = 7 GCs without hBDs and n = 9 GCs with hBDs; in 6 cultures). (D–F) Sample traces of miniature inhibitory postsynaptic currents (mIPSC). Cumulative distribution of the mIPSC amplitudes and mean values of mIPSC amplitude and frequency (n = 8 GCs without hBDs and n = 7 GCs with hBDs; in 4 cultures). No significant difference between granule cells with and without hBDs was observed in these experiments.

Next, we tested whether excitatory synaptic strength is changed in hBDs containing GCs. Individual GCs were patched and miniature excitatory postsynaptic currents (mEPSC) were recorded in whole-cell voltage mode (Figure 2A–C). Although a tendency towards higher mean mEPSC amplitude in hBD-GCs was detected (Figure 2B), this difference did not reach the level of significance (Figure 2C). The mean frequency (no hBDs: 1.89±0.23 Hz; hBDs: 2.03±0.24 Hz), rise time (no hBDs: 2.55±0.14 ms; hBDs: 2.47±0.08 ms) and decay time (no hBDs: 8.01±0.28 ms; hBDs: 8.07±2.55 ms) of mEPSCs were not different between the two groups as well. These results indicated that mEPSC properties are comparable in GCs with and without hBDs in mature organotypic slice cultures.

### Inhibitory Synaptic Strength of hBD-GCs

To assess the strength of inhibitory synapses a different set of cultures was used and miniature inhibitory postsynaptic currents (mIPSC) were recorded from the two GC groups (Figure 2D–F). These recordings revealed no significant difference between hBD and no-hBD-GCs, indicating that inhibitory synaptic strength is comparable between the two types of GCs.

### Dendritic Arborization of GCs with hBDs in the Molecular Layer

**Figure 3:**
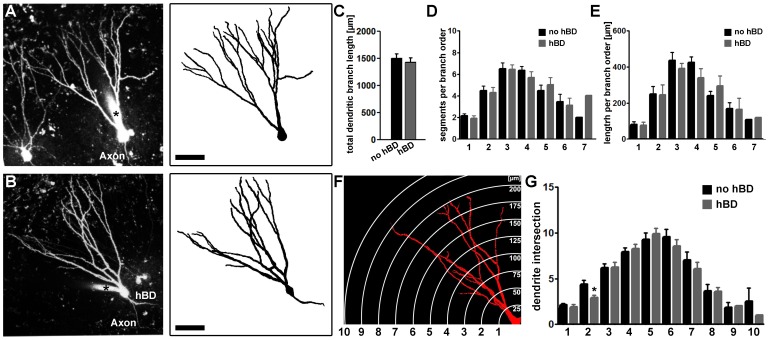
The morphology of the apical dendritic tree of granule cells with hilar basal dendrites. (A, B) Alexa-filled dentate granule cells were reconstructed in confocal image stacks using Neuronstudio software. 2D-projected image stacks (left) and the corresponding reconstructed skeletons (right) are shown for a granule cell without (A) and with a hBD (B). Scale bar: 50 µm. (C–E) No significant difference in total dendritic branch length (TDBL), number of segments per branch order or mean length per branch order was observed between granule cells with and without basal dendrites (n = 21 GCs without hBDs and n = 21 GCs with hBDs; in 12 cultures). (F, G) The Sholl-analysis of the two GC populations showed a comparable complexity of the dendritic trees (distance of circles/spheres in F, 25 µm).

The presence of hBDs distinguishes the two GC types. However, it has not yet been examined in detail in entorhino-hippocampal slice cultures whether the presence of hBDs affects the morphology of the dendritic arbor extending into the molecular layer. Therefore, the dendritic tree within the molecular layer of patched and Alexa568-filled GCs (Figure 3A, B) was reconstructed in 3D-confocal image stacks using Neuronstudio Software [18]. Analysis of the total dendritic branch length (Figure 3C), segment number per branch order (Figure 3D) and mean length per branch order (Figure 3E) revealed no significant difference between the two GC types. A Sholl-analysis did not show any significant difference between the two groups (Figure 3F, G), except for a reduced number of intersections at a distance of 50 µm from the soma in hBD-GCs. The mean length of hBDs was approximately ∼130 µm, i.e., ∼8–10% of the total dendritic branch length (Table 1). We concluded that the dendritic arbor which hBD-GCs extend into the molecular layer is comparable to the dendritic arbor of GCs lacking hBDs in mature entorhino-hippocampal slice cultures.

**Table 1:** Morphological properties of hilar basal dendrites.

	hBD length [µm]	Length per branch order [µm]				Number of segments per branch order			
	total	D1	D2	D3	D4	D1	D2	D3	D4
**mean**	131	56.5	44.9	43.2	43.6	1.21	2.29	2	2
**SEM ±**	16.2	9.1	2.9	3.2	–	0.16	0.29	0	–
**n**	14	14	7	2	1	14	7	2	1

Hilar basal dendrites (hBDs) contribute to ∼8–10% of the total dendritic branch length of hBD-GCs. Most hBD-GCs revealed a single hBD emerging from the basal pole of the soma. In one case two and in another three first order hBD segments were observed. Second order branches were found in 50% of cases. In two cases third order segments and in one case fourth order segments were observed (n = 14 hBD-GCs were analyzed; in 9 cultures).

### Spine Densities of hBD-GCs in the Outer Molecular Layer of the Dentate Gyrus

**Figure 4:**
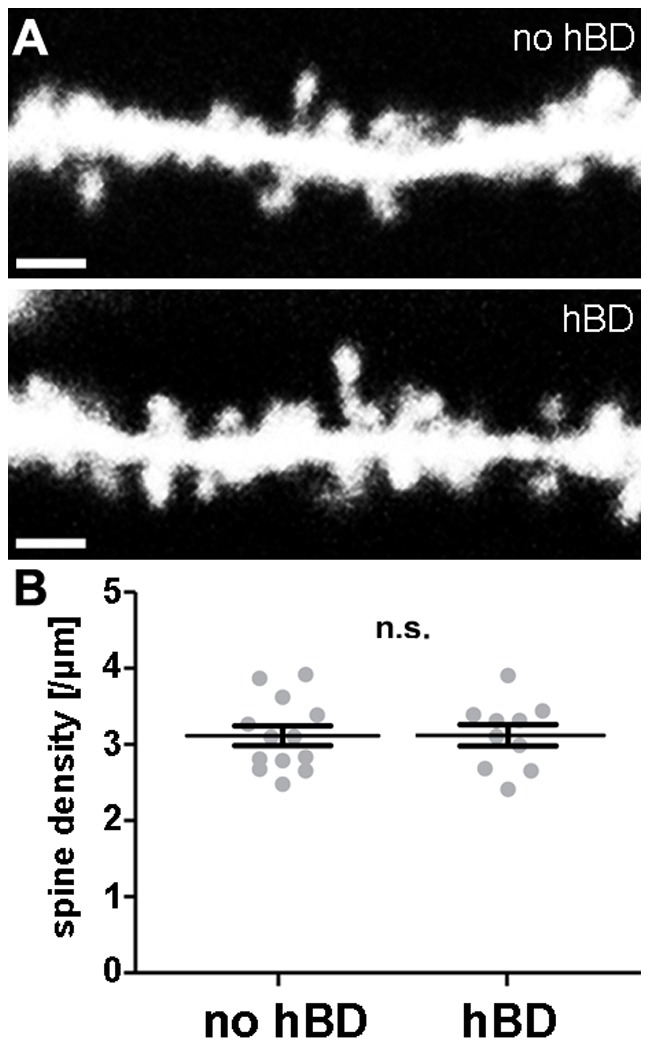
Spine density in the outer molecular layer is not altered in granule cells with hilar basal dendrites. (A) 2D-projected confocal image stacks of individual dendritic segments of dentate granule cells imaged in the outer molecular layer. Scale bar: 2 µm. (B) Spine density analysis of granule cells with and without hBDs (n = 13 segments from no-hBD and 10 segments from hBD-GCs; in 10 cultures). Spine densities were not significantly different.

We also examined spine densities of GCs in the outer molecular layer (OML) of the dentate gyrus, i.e., the layer in which the major input to the hippocampus from the entorhinal cortex terminates. Individual dendritic segments in the OML were imaged at high resolution and the number of spines was analyzed in 3D image stacks [19]. As shown in Figure 4, GCs with and without hBDs were indistinguishable in their mean spine densities in this set of experiments.

### hBD-GCs are not Regularly Targeted by Mossy Fibers of Surrounding GCs in Normal Organotypic Slice Cultures

**Figure 5:**
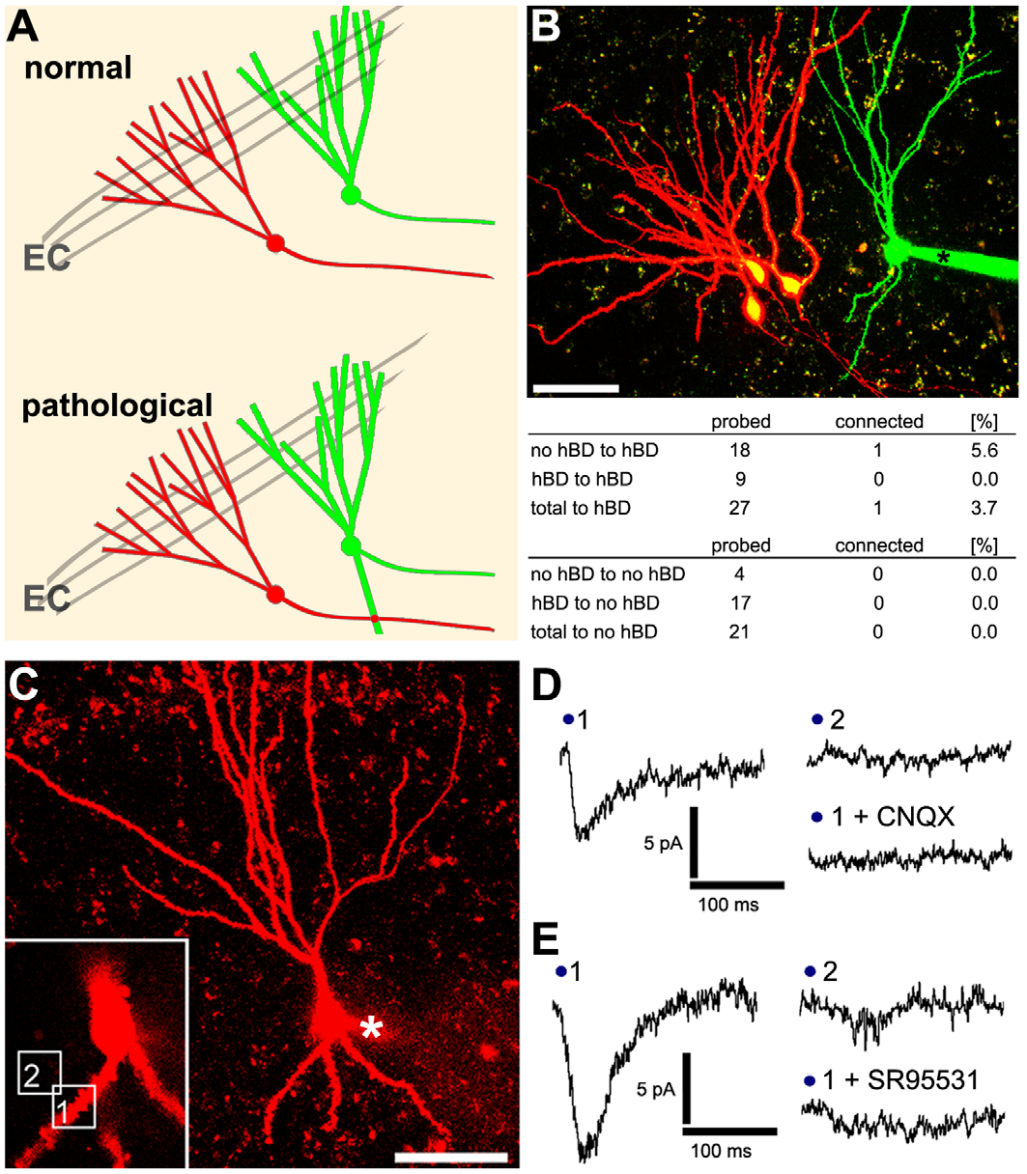
Granule cells with hilar basal dendrites are not heavily innervated by mossy fibers. (A) GC axons, i.e., mossy fibers, contact mossy cells in the hilus and CA3 pyramidal neurons (not shown) without connecting to other GCs. However, under pathological conditions hilar basal dendrites (hBDs) may be innervated by mossy fibers. Computational studies [15] suggests that a strong innervation of hBDs by mossy fibers could promote hyperexcitability in the dentate gyrus network. (B) Paired recordings between neighboring granule cells with and without hBDs revealed no evidence for a heavy GC-to-GC connectivity in the dentate gyrus of mature entorhino-hippocampal slice cultures. The proximity of axons and hBDs was documented in confocal image stacks prior to recordings. In 48 probed connections only one functional connection was found. However, this connection was indeed found on a hBD-GC, thus confirming that functional GC-to-GC connectivity is possible on hBD-GCs (asterisk, patch pipette). Scale bar: 50 µm. (C) Flash photolysis of caged-Glutamate and caged-GABA was performed over hBDs (boxed area 1 in the inset) and in the neuropil in the neighborhood of hBDs (boxed area 2 in the inset) while recording evoked responses from the soma of the same hBD-GC (asterisk, patch pipette). Scale bar: 50 µm. (D, E) Sample traces for GABA and Glutamate uncaging experiments. Evoked responses were blocked by the AMPA-receptor antagonist CNQX or the GABA_A_-receptor blocker SR95531 respectively (in 3 independent experiments each).

It has been suggested that the presence of hBD-GCs could lead to strong GC-to-GC connections in the dentate gyrus, which could promote hyperexcitability under pathological conditions ([5], [15]; Figure 5A). To test whether hBD-GCs are heavily innervated by other GCs in mature entorhino-hippocampal slice cultures under control condition neighboring GCs were patched simultaneously. 50 consecutive APs were induced in one of the two GCs at 0.1 Hz while recording from the other (up to three presynaptic cells probed per postsynaptic partner; Figure 5B). In 48 connections probed total between GCs (both with and without hBDs), a single connection was found on a hBD-GC (overall probability of connections onto hBD-GCs in these experiments ∼5–6%; Figure 5B). We concluded that hBDs-GCs are not regularly innervated by mossy fibers of surrounding GCs in mature entorhino-hippocampal slice cultures.

### Flash Photolysis of Caged-Glutamate and Caged-GABA Suggests the Presence of Functional Postsynaptic Receptors on hBDs of GCs

To exclude the possibility that the inability to detect a strong GC-to-GC connectivity in our slice cultures resides in the lack of functional postsynaptic receptors on hBDs, flash photolysis of caged-Glutamate was performed in the proximity of hBDs, while recording inward currents from the soma of the respective hBD-GC (Figure 5C). Notably, we were able to detect dendritic spines on hBDs (spine density: 2.5±0.1/µm), which suggested the presence of functional excitatory postsynapses. Indeed, a response to the photolysis of caged-Glutamate could be evoked at hBDs. Likewise, GABA-uncaging induced corresponding inward currents at hBDs (different set of hBD-GCs recorded with high chloride internal solution). The evoked currents were blocked by the application of CNQX or SR95531, respectively (Figure 5D, E), and did not occur when the laser beam was directed onto the neuropil in the neighborhood of hBDs or onto axons (at comparable distances from the soma; 20–30 µm). We concluded that AMPA-receptors and GABA-receptors can be activated on hBDs in mature entorhino-hippocampal slice cultures.

## Discussion

In the present study we compared structural and functional properties of GCs with and without hBDs in a mature organotypic environment under control conditions. Our results show that hBD-GCs and GCs lacking hBDs are very similar in their functional and structural properties. Thus, in control cultures the presence of hBDs on GCs is neither sufficient to predict alterations in the basic functional and structural properties of these GCs nor indicative of a heavy GC-to-GC connectivity between neighboring GCs.

### Entorhino-hippocampal Slice Cultures - a Useful Tool to Study Functional and Structural Properties of Dentate Granule Cells

Entorhino-hippocampal slice cultures recapitulate major steps of hippocampal development and show a neuronal connectivity similar to the in vivo situation [19]–[25]. Nevertheless, they cannot be directly compared to an adult in vivo hippocampus. For example, extrinsic afferents such as the septo-hippocampal projection, commissural axons or crossed entorhinal projections are absent. Regardless of this consideration, organotypic hippocampal slice cultures have been widely used to understand cellular mechanisms and/or aspects of neuronal organization in the hippocampus [26]–[29]. Their structural integrity can be easily monitored prior to recordings and structural and functional analysis can be combined with pharmacological, molecular biology and imaging approaches (e.g., [24]). GCs are well-preserved in these cultures (e.g., [30], [31]) and electrophysiological recordings from GCs are very robust (e.g., [23]). Thus, organotypic slice cultures appear to be appropriate preparations to study basic aspects of granule cell function and structure.

Slice cultures are usually prepared at an early age (at less than 7 days postnatally; [32]). They mature during the in vitro cultivation period and become stable. As has been demonstrated earlier [33], [34], spines and synapses stabilize during the second and third week in vitro. This is in line with our own work, in which we could show that spine densities and mEPSC properties of GCs are stable over days and weeks in ≥18 div old slice cultures [19], [23]. Taken together, these data indicate that near to steady-state conditions are reached in cultures after ∼2.5 weeks of in vitro differentiation. Under these steady-state conditions, it is possible to investigate and compare the properties of neighbouring GCs with and without hBDs, which have developed in the same set of cultures.

In other studies, acute slice preparations or fixed slices of adult animals have been used to analyze GCs and hBD-GCs (e.g., [8], [12], [13], [35]–[37]). These methods have their own advantages and disadvantages, such as the acute slicing of the hippocampus, which leads to an acute denervation of GCs and, possibly, to structural and functional changes [19], [23], [38], [39]. The entorhino-hippocampal slice culture preparations used in our study are not intended to replace these techniques. Rather, the slice cultures employed here are an additional and in our eyes highly suitable way to study GC and hBD-GC function-structure relationships. The data reported in this study provide a baseline for future work focusing on the role of hBD-GCs in organotypic slice cultures.

### Dentate GCs with hBDs do not Differ in their Major Functional and Structural Properties from GCs without hBDs in Control Cultures

While we were not able to detect any major difference between GCs with and without hBDs with the techniques employed in the present study, and both GC-types were essentially indistinguishable (with the exception of a minor difference in the proximal dendritic tree and the presence of hBDs), we cannot exclude that more subtle differences may have escaped our detection. For example dual somato-dendritic patch clamp recordings and computational approaches may be required to precisely evaluate the functional properties of individual dendritic segments [21], [22]. In these studies it was shown that the dendrites of GCs exhibit mainly passive properties. It remains unknown, whether the ‘apical’ dendrites of hBD-GCs or even the hBDs themselves could differ in their functional properties from ‘regular’ GC-dendrites. Moreover, we cannot exclude the possibility that in this context the slight difference observed in our Sholl-analysis, i.e., reduced complexity of the proximal part of the dendritic tree, could have an effect on dendritic integration in hBD-GCs. With these considerations kept in mind, we nevertheless conclude with confidence from our data that hBD-GCs and GCs without hBDs in vitro do not show major differences in their basic functional and structural properties.

### GCs with hBDs do not Show a Strong GC-to-GC Connectivity in Control Cultures

It has been suggested that hBDs extending from GCs into the hilus of the dentate gyrus could be targets for the mossy fibers of other GCs [5], [13]. Thus, GC-to-GC synapses could form an excitatory recurrent loop, which could increase the overall excitability of the dentate gyrus; even without changes in other functional or structural properties of GCs [40]. In an elegant computational study Morgan and Soltesz [15] have shown that a small percentage of highly interconnected GCs, presumably hBD-GCs, could indeed affect the excitability of the otherwise remarkably robust dentate gyrus network.

Since hBD-GCs exist under control conditions in vitro [7] as well as in vivo [8], we wondered whether these dendrites receive their major functional input from mossy fibers, i.e., whether hBDs are indicative of normally occurring recurrent GC-to-GC connections. To address this issue, we attempted to find GC-GC pairs in our slice culture preparations and patched GCs simultaneously. We reasoned that the chance to detect GC-to-GC connections should be particularly high between neighboring GCs, since mossy fibers emerge from the basal pole of the GC soma and extend into the hilar region, i.e., the region in which hBDs of adjacent GCs can be found. Indeed, proximity of axons and hBDs of the patched GCs could be visualized in confocal image stacks in these experiments (see Figure 5B). Although it is not possible to fully exclude the possibility that long-distance and/or ‘silent’ [41]–[43] connections between GCs may exist, the data clearly show that under control conditions hBD-GC are not functionally innervated by the majority of surrounding GCs in mature entorhino-hippocampal slice cultures.

The inability to detect a strong GC-to-GC innervation in these experiments was not due to the lack of functional receptors on hBDs as revealed by flash photolysis of caged-Glutamate. In line with this observation dendritic spines were regularly observed on hBDs. GABA_A_-R mediated inward currents could be evoked on hBDs as well, suggesting that hBDs receive inhibitory inputs. The presynaptic neurons which terminate on the hBDs in mature entorhino-hippocampal slice cultures are not known and need to be identified. Our data suggest, however, that the majority of excitatory synapses formed on hBDs are unlikely to be functional synapses formed by mossy fibers. We conclude that the presence of hBDs *per se* cannot be trivially interpreted as a sign of a strong excitatory GC-to-GC connectivity in the normal dentate gyrus in vitro.

### Dentate Granule Cells with hBDs may form Aberrant Networks Under Pathological Conditions

The GCs investigated in the present study were all from mature and untreated control cultures. These GCs may differ from GCs observed under pathological conditions (e.g. [8], [12], [36], [44]). One major difference could be the length of the hBDs extended into the hilus. For example, the mean length of hBDs observed in our cultures was ∼130 µm. Although this is in line with previous studies reporting lengths between 20 µm and 130 µm in pilocarpin models of epilepsy [13], [45], hBDs with an average length of ∼600 µm have also been reported [37]. These long hBDs could receive more than 10% of the inhibitory and excitatory input of the GCs [37] including numerous aberrant GC-to-GC connections.

Since mature granule cells remain plastic neurons, which are able to remodel their dendrites and their connectivity, for example following entorhinal denervation [19], [46], [47], it is conceivable that hBD-GCs may elongate their basal dendrites under pathological conditions and/or could form new synapses with mossy fibers. Likewise, newly formed GCs could play a role in this process: Since GCs are continuously generated in the dentate gyrus and integrated into the dentate gyrus network [48]–[51], hBDs could not only persist on immature GCs [7] but even elongate and/or form aberrant GC-to-GC connections under pathological conditions. This suggestion is supported by a recent study that showed aberrant circuit formation on hBDs of immature GCs in a hypoxia model of epilepsy [52]. Thus, hBD-GCs, which seem to be functionally normal under control conditions (this study), could turn into GCs with altered functional/structural properties and/or into the “hubs” of a recurrent excitatory network, as has been suggested by others [15]. Accordingly, “harmless” hBD-GCs could change their role and could become important players in the pathogenesis of hippocampal epilepsy.

## Materials and Methods

### Ethics Statement

Animal care and experimental procedure were performed in agreement with the German law on the use of laboratory animals (animal welfare act; TierSchG; §4 Abs. 3) and approved by the animal welfare officer of Goethe-University, Faculty of medicine (reference number BB01/10/2011).

### Preparation of Slice Cultures

Entorhino-hippocampal slice cultures were prepared at postnatal day 4–5 from C57BL/6J mice of either sex using a published protocol [32]. No attempt was made to distinguish between sexes in the experiments. Cultivation medium contained 50% (v/v) MEM, 25% (v/v) basal medium eagle, 25% (v/v) heat-inactivated normal horse serum, 25 mM HEPES buffer solution, 0.15% (w/v) bicarbonate, 0.65% (w/v) glucose, 0.1 mg/ml streptomycin, 100 U/ml penicillin, and 2 mM glutamax. The pH was adjusted to 7.3 and the medium was replaced every second day. All slice cultures were allowed to mature for 18–20 days in humidified atmosphere with 5% CO_2_ at 35°C.

### Whole-cell Patch-clamp Recordings

Whole-cell patch-clamp recordings from dentate granule cells were carried out at 35°C as previously described [23]. The bath solution contained 126 mM NaCl, 2.5 mM KCl, 26 mM NaHCO_3_, 1.25 mM NaH_2_PO_4_, 2 mM CaCl_2_, 2 mM MgCl_2_, and 10 mM glucose and was bubbled with 95% O_2_/5% CO_2_. For paired recordings, input-output curves and mEPSC recordings patch pipettes contained 126 mM K-gluconate, 4 mM KCl, 4 mM ATP-Mg, 0.3 mM GTP-Na_2_, 10 mM PO-Creatine, 10 mM HEPES, 20 µM Alexa568 or Alexa488 and 0.3% Biocytin (pH = 7.25 with KOH, 290 mOsm with sucrose). mEPSC recordings were carried out at a holding potential of −70 mV in the presence of 10 µM D-AP5, 10 µM SR-95531 and 0.5 µM Tetrodotoxin (TTX). For mIPSC recordings patch pipettes contained 40 mM CsCl, 90 mM K-gluconate, 1.8 mM NaCl, 1.7 mM MgCl_2_, 3.5 mM KCl, 0.05 mM EGTA, 2 mM ATP-Mg, 0.4 mM GTP-Na_2_, 10 mM PO-Creatine, 10 mM HEPES, 20 µM Alexa568 and 0.3% Biocytin (pH = 7.25 with KOH, 290 mOsm with sucrose); dentate granule cells were recorded at −70 mV in the presence of 10 µM D-AP5, 10 µM CNQX and 0.5 µM TTX. Series resistance was monitored in 2–3 min intervals and recordings were discarded if the series resistance was >30 MΩ. For I-V curves series resistance was determined prior and after the recordings and data were discarded if series resistance was >15 MΩ and changed during the recording >10%.

### Imaging of Dentate Granule Cells

Patched granule cells were filled with Alexa568 or Alexa488 (20 µM) and visualized using a Zeiss LSM Exciter confocal microscope (40× water immersion objective lens; 0,8 NA; Zeiss) and 1× scan zoom. Individual dendritic segments in the outer molecular layer or hBDs were visualized using 4× scan zoom [19].

### Paired Recordings

To estimate the functional connectivity between granule cells under control conditions in mature entorhino-hippocampal slice cultures, neighboring GCs were simultaneously patched in regular external solution. The identity of GCs (no-hBD or hBD) was determined in confocal image stacks prior to recordings. 50 consecutive action potentials were elicited at 0.1 Hz in the presynaptic cell while recording from the postsynaptic neuron. The connectivity of up to three GCs (∼20–70 µm apart from the soma of the postsynaptic cell) was probed on GCs (both with and without hBDs).

### Flash Photolysis of Caged-Glutamate and Caged-GABA

For local stimulation of hBDs hBD-GCs were patch-clamped in the presence of Rubi-Glutamate (40 µM) or Rubi-GABA (10 µM; both from Tocris Bioscience, UK, [53], [54]) in TTX (0.5 µM)-containing bath solution. The microscope was focused over the maximal cross-sectional area of an individual hBDs at a distance of 20–30 µm from soma. A region of interest (8×8 µm^2^) was selected containing the hBD and flash photolysis was performed using the bleaching function of Zeiss Zen software (AOTF-controlled Argon laser 488 nm; 100% transmission; single bleach iterations, <1 ms duration). Uncaging was performed 20 times at 0.1 Hz while recordings evoked inward currents at the soma. Uncaging in the neuropil in the neighborhood of the hBDs or on top of axons served as controls in these experiments (similar distance from soma). In some experiments AMPA-receptor blockers (CNQX; 50 µM) and GABA-receptor blockers (SR95531; 10 µM) were added to the bath solution while uncaging Glutamate or GABA to assure the specificity of the evoked response.

### Quantifications and Statistics

#### Reconstruction of the dendritic tree

The dendritic tree of individual patch-clamped and Alexa-filled dentate granule cells with and without basal dendrites was manually reconstructed in confocal image stacks using Neuronstudio 0.9.92 (CNIC Mount Sinai School of Medicine, NY; [18]). The mean numbers of segments per branch order as well as the mean length per branch order were determined using the reconstructed skeleton file. Total dendritic branch length (TDBL) was defined as the sum of all branch lengths of individual dendritic trees.

#### Sholl sphere analysis

The “Fiji”-plugin “Simple neurite Tracer” [55], download from: http://fiji.sc/) was used to assess and compare the complexity of dendritic trees between the two GC-groups. For Sholl-analysis the soma was chosen as the centerpoint and a circle/sphere separation of 25 µm was used (standard axes and no normalisation of intersections).

#### Spine density

Dendritic spines were assessed manually on 3D-image stacks of dendritic segments using the Zeiss LSM image browser to navigate through the stacks as described [19]. All dendritic protrusions were counted as dendritic spines, regardless of their morphological characteristics. Images were analyzed blind to experimental condition to ensure unbiased observation. For each segment a defined distance (∼30 µm) from a dendritic branch point was analyzed and all spines were counted. Spine density (spines per µm dendrite) was calculated based on these results.

#### Electrophysiology

Electrophysiological data were assessed using pClamp 10.2 (Axon Instruments, USA), MiniAnalysis (Synaptosoft, USA) and MATLAB v.7.5 (The MathWorks Inc., Natick, MA, USA) software. Miniature events were visually inspected and detected by an investigator blind to experimental condition with MiniAnalysis software. 300–400 miniature postsynaptic events were inspected per recorded neuron. I-V curves were analyzed with a custom-made script written in MATLAB.

#### Statistics

Statistical comparisons were made using the nonparametric Wilcoxon-Mann-Whitney test. P-values of less than 0.05 were considered to be a significant difference. All values are expressed as mean ± standard error of the mean (SEM).

### Digital Illustrations

Confocal image stacks were exported as 2D-projections from the Zeiss LSM image browser and stored as TIFF files. Figures were prepared using Photoshop CS2 graphics software (Adobe, San Jose, CA, USA), Inkscape (Free Software Foundation, Boston, MA, USA; download from: http://inkscape.org/) and GraphPad Prism 5 (GraphPad Software, San Diego, USA). Image brightness and contrast were adjusted.
